# Carbapenem-resistant Pseudomonas aeruginosa with acquired bla(vim) metallo-beta-lactamase determinants, Italy.

**DOI:** 10.3201/eid0603.000314

**Published:** 2000

**Authors:** G M Rossolini, M L Riccio, G Cornaglia, L Pagani, C Lagatolla, L Selan, R Fontana


### Carbapenem-Resistant *Pseudomonas aeruginosa* with Acquired *bla*$_{VIM}$ Metallo-ß-Lactamase Determinants, Italy

**To the Editor:** Acquired metallo-ß-lactamase determinants in ***Pseudomonas aeruginosa*** and other major bacterial pathogens are of concern for development of antimicrobial drug resistance. The carbapenemase and extended-spectrum cephalosporinase activity of metallo-ß-lactamases, as well as their resistance to ß-lactamase inhibitors, may severely limit the antimicrobial agents active against bacterial strains that produce such enzymes (1,2). Antimicrobial chemotherapy may become ineffective against ***P. aeruginosa*** strains with a multidrug-resistant phenotype that have acquired a metallo-ß-lactamase determinant.

We recently described a new acquired metallo-ß-lactamase determinant, ***bla***$_{VIM}$, in a carbapenem-resistant ***P. aeruginosa*** clinical isolate (VR-143/97) from the University Hospital of Verona, Italy (3). This isolate was the index strain of an outbreak of ***bla***$_{VIM}$-positive ***P. aeruginosa***, which was caused both by strains that were clonally related to VR-143/97 and by clonally unrelated strains (4). ***bla***$_{VIM}$ is the second known metallo-ß-lactamase determinant that can spread among ***P. aeruginosa***; the first was ***bla***$_{IMP}$, which was detected in the early 1990s in nosocomial isolates of various ***Enterobacteriaceae***, ***P. aeruginosa,*** and other nonfastidious gram-negative nonfermenters from the Far East (1,2,5-7) and, recently, in an ***Acinetobacter baumannii*** clinical isolate from Italy (8). Although completely unrelated at the sequence level, ***bla***$_{VIM}$ resembles ***bla***$_{IMP}$ in being carried on an integron-borne mobile gene cassette and in encoding an enzyme (VIM-1) with broad substrate specificity (3). Because of these properties, ***bla***$_{VIM}$ has the potential to become a dangerous resistance determinant.

An analysis of carbapenem-resistant ***P. aeruginosa*** from Italian hospitals since 1998 showed production of metallo-ß-lactamase, assayed as described (3), in five isolates from three hospitals in Italy. Two isolates (PPV-97 and PPV-108) were from the University Hospital of Pavia (PPV-97 was isolated in September 1998 from the urine of an inpatient in the neurosurgery department, and PPV-108 was isolated in November 1998 from a decubitus ulcer of an inpatient in the vascular surgery department); two (TS-832035 and TS-832347) were isolated in February 1999 from the University Hospital of Trieste (both from the blood of inpatients, in the intensive care unit and in the internal medicine department, respectively); and one (SAP-01/99) was isolated in September 1999 from the Rome University Hospital "Policlinico Umberto I" (from the blood of an inpatient in the vascular surgery department). Except for those from Pavia Hospital, where the two departments share the same surgical unit, no epidemiologic relationship could be established among any of them or with those previously isolated in Verona (3,4).

The five isolates were highly resistant to carbapenems (MICs for imipenem and meropenem were >64 µg/mL) and to carbenicillin, ticarcillin, ticarcillin/clavulanate, piperacillin, piperacillin/tazobactam, mezlocillin, ciprofloxacin, gentamicin, tobramycin, and netilmicin. All five were also resistant to ceftazidime and cefepime, except for SAP-01/99, which had intermediate resistance to the above drugs. Some isolates retained susceptibility to aztreonam (PPV-97, TS-832035, and SAP-01/99) or amikacin (PPV-108, TS-832035, and TS-832347).

In a colony-blot hybridization assay (3), all the above isolates were recognized by a ***bla***$_{VIM}$-specific probe consisting of an amplicon that contained the entire ***bla***$_{VIM}$ coding sequence (3). None were recognized by a probe specific for ***bla***$_{IMP}$ and consisting of a 0.5-kb ***Hin***dIII fragment from the ***bla***$_{IMP}$ gene (8).

Our results indicate that circulation of carbapenem-resistant ***P. aeruginosa*** carrying ***bla***$_{VIM}$ metallo-ß-lactamase determinants, originally detected in one Italian hospital (3-4), could soon become widespread. The detection in different hospitals of ***bla***$_{VIM}$-positive isolates that apparently were epidemiologically unrelated suggests that the environmental reservoir of ***bla***$_{VIM}$-containing strains is relatively broad and that this novel determinant has potential relevance for the emerging phenomenon of carbapenem resistance in ***P. aeruginosa***. Since we did not sequence the ***bla***$_{VIM}$-related genes carried by the various isolates, we do not yet know whether they differ from that cloned from ***P. aeruginosa*** VR-143/97 (3). The five isolates described in this report are being characterized to ascertain their clonal relatedness and identify the sequences of their ***bla***$_{VIM}$-related determinants. The recent appearance of this and other acquired metallo-ß-lactamases among ***P. aeruginosa***

and other gram-negative pathogens in Europe (8-10) underlines the need for systematic surveillance to monitor the spread of similar resistance determinants.

This work was supported by grants no. FMRX-CT98-0232 from the European TMR Research Network on metallo-ß-lactamases and no. 9906404271 from MURST ex-40%.

**Gian Maria Rossolini,* Maria Letizia Riccio,* Giuseppe Cornaglia,† Laura Pagani,‡ Cristina Lagatolla,§ Laura Selan,¶ and Roberta Fontana†**
* Università di Siena, Siena, Italy; †Università di Verona, Verona, Italy; ‡Università di Pavia, Pavia, Italy; §Università di Trieste, Trieste, Italy; and ¶Università "La Sapienza," Rome, Italy

### References

1. Livermore DM. Acquired carbapenemases. J Antimicrob Chemother 1997;39:673-6.
2. Rasmussen BA, Bush K. Carbapenem-hydrolyzing ß-lactamases. Antimicrob Agents Chemother 1997;41:223-32.
3. Lauretti L, Riccio ML, Mazzariol A, Cornaglia G, Amicosante G, Fontana R, et al. Cloning and characterization of ***bla***$_{VIM}$, a new integron-borne metallo-ß-lactamase gene from a ***Pseudomonas aeruginosa*** clinical isolate. Antimicrob Agents Chemother 1999;43:1584-90.
4. Mazzariol A, Cornaglia G, Piccoli P, Lauretti L, Riccio ML, Rossolini GM, et al. Carbapenem-hydrolyzing ß-lactamases in ***Pseudomonas aeruginosa***. Eur J Clin Microbiol Infect Dis 1999;18:455-6.
5. Senda K, Arakawa Y, Nakashima K, Ito H, Ichiyama S, Shimokata K, et al. Multifocal outbreaks of metallo-ß-lactamase-producing ***Pseudomonas aeruginosa*** resistant to broad-spectrum ß-lactams, including carbapenems. Antimicrob Agents Chemother 1996;40:349-53.
6. Lee K, Chong Y, Shin HB, Yong D. Rapid increase of imipenem-hydrolyzing ***Pseudomonas aeruginosa*** in a Korean hospital. In: Program and Abstracts of the 38th Interscience Conference on Antimicrobial Agents and Chemotherapy. Washington: American Society for Microbiology; 1998. [Abstract E85].
7. Koh TH, Babini GS, Woodford N, Sng LH, Hall LM, Livermore DM. Carbapenem-hydrolysing IMP-1 ß-lactamase in ***Klebsiella pneumoniae*** from Singapore. Lancet 1999;353:2162.
8. Cornaglia G, Riccio ML, Mazzariol A, Lauretti L, Fontana R, Rossolini GM. Appearance of IMP-1 metallo-ß-lactamase in Europe. Lancet 1999;353:899-900.
9. Woodford N, Palepou M-FI, Babini GS, Bates J, Livermore DM. Carbapenemase-producing ***Pseudomonas aeruginosa*** in the UK. Lancet 1998;352:546-7.
10. Cardoso O, Sousa JC, Leitâo R, Peixe L. Carbapenem-hydrolysing ß-lactamase from clinical isolates of ***Pseudomonas aeruginosa*** in Portugal. J Antimicrob Chemother 1999;44:135.

### Malaria and Global Warming in Perspective?

**To the Editor:** I read with great interest the article "From Shakespeare to Defoe: malaria in England in the Little Ice Age" (1). Unfortunately, the article is not as balanced as a presentation last year by Paul Reiter, which clearly illustrated that, although climate is important in the transmission of malaria, the influence of other factors (e.g., access to medical care and improved housing) is likely to be of more importance in Europe.

Malaria indeed was quite common in Europe, even in the Roman Empire and in Medieval Europe, and until a few decades ago, it was still present in parts of Europe, Australia, and North America. In fact, the failure of the 1806 British invasion of Zeeland in the Netherlands may be attributable to infection of the British forces with malaria. However, the authors referenced by Reiter have never made the claim that in the coming years warmer "temperatures will result in malaria transmission in Europe and North America." On the contrary, the reports of the Intergovernmental Panel on Climate Change Reiter quotes conclude that "Although climate change could increase the potential transmission of malaria [in Europe and North America], existing public health resources—disease surveillance, surface water management, and treatment of cases—would make reemergent malaria unlikely" (2,3).

Reiter's argument that some scientists attribute the recent observed increase in malaria risk to climate trends is also not accurate. While acknowledging the sensitivity of the malaria mosquito and parasite to climate, these researchers examine insect and incidence data to explore multiple factors underlying malaria emergence. Another group of scientists uses mathematical simulation models to estimate changes in malaria risk over the next few decades. These models, which are heuristic tools not meant to predict future worlds, assess how potential risk for malaria may by affected by changes in climate (4). The goals of both types of research are to improve knowledge of the complex malaria transmission cycle, define epidemic-prone areas, identify the reasons for increased malaria risk, and develop solutions to protect vulnerable communities.

